# Integrating multi-omics and clinical features to model survival in epithelial ovarian cancer subtypes

**DOI:** 10.1038/s41598-025-29403-6

**Published:** 2025-12-19

**Authors:** Raghda E. Eldesouki, Omar Tarek, Hassan Morsi

**Affiliations:** 1 Genetics Unit, Department of Histology and Cell Biology, School of Medicine, Suez Canal University, Ismailia, Egypt; 2 Faculty of Engineering, Suez Canal University, Ismailia, Egypt; 3 Department of Obstetrics and Gynaecology, School of Medicine, Ain Shams University, Cairo, Egypt

**Keywords:** Epithelial ovarian cancer (EOC), Serous cystadenocarcinoma (SC), Endometrioid adenocarcinoma (EA), Machine learning, Integrative analysis, Survival prediction, Gene expression, Molecular signatures, Prognostic biomarkers, The Cancer Genome Atlas (TCGA), Computational biology and bioinformatics, Medical research, Molecular medicine

## Abstract

Epithelial ovarian cancer (EOC) exhibits significant heterogeneity in clinical outcomes, influenced by histology, age, stage, and molecular characteristics. This study aimed to develop and validate a comprehensive model integrating demographic, clinical, and molecular data from The Cancer Genome Atlas (TCGA) to predict two-year survival outcomes in EOC. The cohort included 2,427 patients with Endometrioid Adenocarcinoma (EA)s and Serous Cystadenocarcinoma (SC) , of whom 1,011 had gene data. Machine learning models, including Logistic Regression, Gradient Boosting Classifier (GBC), Support Vector Machines (SVM), and Random Forest, were trained and evaluated for predictive performance. SVM provided the optimal balance of mortality-class detection and overall performance. While GBC achieved the highest ROC-AUC (0.81), SVM demonstrated superior recall for mortality cases (0.70 vs. 0.61), which was prioritized given our clinical objective. Shapley Additive Explanations (SHAP) analyses revealed that WT1, HOXA11, TPM4, TMPRSS2, MUC16, SDHD, and MYC were the most influential predictors of mortality, along with age at diagnosis. Differential gene expression and enrichment analyses identified distinct age- and stage-associated molecular profiles, with genes involved in cell cycle regulation, tumor microenvironment, and growth factor signaling showing significant upregulation. Mutational analyses revealed histology-specific patterns, with TP53, PIK3CA, and ZFHX3 highly mutated in SC, while PTEN and ARID1A were more prevalent in EA. Several mutations, including TP53, FAT3, and FAT4 in EA, and CSMD3 in SC, were associated with poorer survival. Integrating multivariate predictive modeling with biological interpretation provides a comprehensive framework for personalized risk stratification and treatment decision-making in EOC. The identified prognostic biomarkers, such as TPM4, SDHD, MUC16, and BCL6, represent potential targets for future studies and therapeutic interventions.

**Supplementary Information:**

The online version contains supplementary material available at 10.1038/s41598-025-29403-6.

## Introduction

Epithelial ovarian cancer (EOC) is one of the most prevalent gynecological malignancies and continues to carry a high disease burden globally. Previously reported prognostic factors used to include histopathological classification, tumor grade, and FIGO stage^1^. While these clinical and pathological features provide important information, they are insufficient for precise, individualized survival prediction.

In recent years, machine learning (ML) has gained traction as a powerful tool for survival prediction and clinical decision support in EOC. A range of models, including gradient boosting, random forests, support vector machines, and deep learning approaches such as DeepSurv, have consistently outperformed traditional regression-based methods, showing superior accuracy and calibration in both internal and external validation cohorts^2–7^. These models have been applied to diverse data sources—ranging from demographic and clinical variables to imaging-derived features such as radiomics and ultrasound data—demonstrating utility in predicting overall survival, recurrence-free survival, platinum resistance, and treatment response^8–12^. More recently, multimodal approaches combining clinical, radiologic, and histopathological data have further improved risk stratification in high-grade serous ovarian cancer^13^. Despite the the efforts of applying machine learning models, up to our knowledge, there have been no specific studies that integrate demographic, clinical, and molecular features for individualized survival prediction. This study aimed to develop and validate a comprehensive model integrating the forementioned parameters. The Cancer Genome Atlas (TCGA) to predict two-year survival outcomes. Additionally, complementary survival, gene expression, and enrichment analyses were conducted to provide model interpretability and identify molecular signatures associated with prognosis. By merging predictive accuracy with biological insights, this work aims to offer interpretable predictions for patient care and generate biological insights for further research.

## Methodology

### Data source and features selection

Data were sourced from The Cancer Genome Atlas (TCGA) via the GDC Data Portal (https://portal.gdc.cancer.gov/; accessed November 30, 2024). The study included only cases of serous cystadenocarcinoma (SC, morphology code 8441/3) and endometrioid adenocarcinoma (EA, morphology code 8380/3), totaling 2,427 cases, in alignment with SEER morphology classifications (U.S. National Cancer Institute SEER Training Modules, Tumor Morphology | SEER Training https://www.training.seer.cancer.gov/ovarian/abstract-code-stage/ovarian-morphology.html). All data collection adhered to TCGA publication and access policies, and no additional local ethical approval was necessary since only publicly available de-identified data were utilized. For model development, we integrated demographic, clinical, and molecular data. The clinical and demographic features analyzed included vital status, age at index, primary diagnosis, tumor morphology, tumor classification, race, treatment type, and FIGO stage. Additionally, a derived feature, years with disease, was calculated as the difference between age at death and age at diagnosis. The initial dataset comprised 2,427 samples with available information for the two subtypes (Dataset 1). Out of this dataset, a total of 1011 samples had available gene data (Figure S1, Table S1, Dataset 2). For the treatment type feature, input with both “Yes” and “No” entries were resolved by setting the value to “Yes” if it appeared. Placeholders like “—” and “unknown” were replaced with **np.nan** to facilitate the handling of missing data. The tumor feature classification was omitted due to a high percentage of missing or unknown values. The target variable, vital status, was converted into a binary format, with “alive” assigned a value of 0 and “dead” a value of 1. The primary diagnosis column frequently included composite values (e.g., “NOS, Endometrioid, Adenocarcinoma” in a single record); to manage this, the scikit-learn multilabel binarizer was used, creating multiple binary features to represent each diagnosis category. Most features had minimal missing data, except for age at index, which had about 15% missing values for which we used Multiple Imputation by Chained Equations (MICE)^14^. Finally, the gene data were consolidated into 2427 data points using an inner join.

#### Data Cleaning and Preparation

Following the data cleaning process, which involved addressing duplicate entries and eliminating irrelevant features, the final dataset was prepared for modeling. The resultant data frame comprised 944 rows (dataset 3) and 141 features total, comprising clinical, demographic, and gene expression variables.

#### Data splitting

The dataset was divided into training and testing sets using a standard 80/20 split. To ensure consistent class distribution and reduce sampling bias, the split was stratified based on the target variable.

#### Model training

Four classification models were evaluated, including Logistic Regression, Gradient Boosting Classifier (GBC), Support Vector Machines (SVM), and Random Forest. For hyperparameter optimization, scikit-learn’s GridSearch CV was used for each model. The search was configured to employ 5-fold cross-validation, ensuring a robust performance evaluation. The primary goal of the grid search was to find the hyperparameter combination that maximized the F1-score, as this metric was considered most crucial for the study’s objectives. Mortality-class recall and F1-score were pre-specified as primary metrics, with ROC-AUC as secondary. Chi-squared tests were used for categorical features, and Interquartile Range (IQR) or Z-score methods were applied for numerical features based on their distribution, ensuring only relevant and reliable features were retained for the GBC.

#### Model explainability

To clarify the factors influencing the models’ predictions, Shapley Additive Explanations (SHAP) values were calculated after training. This analysis quantified the contribution and importance of each gene in predicting the target classes, thereby offering critical insights into the underlying biology.

### Scaling and normalization

We employed two scaling methods: the standard scaler and the robust scaler. The robust scaler was specifically applied to the gene data to address its non-normal distribution and the presence of outliers. Although we considered using a log scale, we refrained from doing so because some genes have negative values. To gain a deeper understanding of the data, we conducted principal component analysis with varying numbers of components. Approximately 81 features were necessary to account for 95% of the variance.

### Heatmaps and genetic profiling

Clustered heatmaps were created for patients with available expression and survival data using the Coolworm Python library. Gene expression values were normalized by Z-score on a column-wise basis. Hierarchical clustering was applied to both genes and samples. These heatmaps visualized co-regulated genes and expression-based patient subgroups, underscoring their biological and clinical significance. Volcano Plot Analysis Gene expression comparisons were stratified by age (< 62 vs. ≥62 years) and FIGO stage (≤ III vs. > III) within each tumor subtype. A t-test was conducted for each gene, and log2 fold change (FC) along with p-values were calculated. Genes were deemed significant if *p* < 0.05 and |log2FC| > 1. Volcano plots highlighted significant genes in red and non-significant ones in blue, with thresholds clearly marked. Visualization was done using Pandas, NumPy, Seaborn, Matplotlib, and SciPy libraries.

### Enrichment analysis

The disease, function, and pathway enrichment analyses of genes that showed high predictability for death versus alive outcomes, were conducted using the SR plot images GO pathway enrichment analysis (http://www.bioinformatics.com.cn/basic_local_go_pathway_enrichment_analysis_122_en ). Each gene score represents a transformation ( (−log₁₀ p-value)) of the corresponding p-value, with higher scores indicating stronger associations. Genes and their FC which have shown statistically significant expression, in the previous step, were uploaded to the SR plot prompt.

### Mutation–Outcome Correlation Analysis

Mutational effects were analysed by comparing clinical outcomes between **mutated vs. non-mutated** cases for each gene: mean survival outcomes (vital status) were compared using mean-difference analysis. Results were stored as gene–outcome correlation pairs. Python libraries (pandas, numpy) were used for analysis.

### Cox Proportional Hazards Model

A multivariable Cox proportional hazards model was implemented using the lifelines. Cox PHFitter class^15^ to assess the association between genetic mutations and survival outcomes. The model employed “Time” as the duration of survival and “Status” as the event indicator (1 = dead, 0 = alive). The duration of survival was calculated based on the difference between the date of death and date of diagnosis. Results, including hazard ratios and statistical significance, were obtained using the library lifelines. A forest plot of the hazard ratios was created with the matplotlib library to illustrate the contribution of each genetic variable to survival risk^16^.

### Kaplan–Meier survival analysis

Survival curves were generated for selected mutations using Kaplan Meier Fitter in lifelines. Survival probabilities were compared between mutation-present vs. mutation-absent groups for the top 50 genes.

## Results

### Cohort description

In the training dataset (*n* = 2,427) and the subset with gene data (*n* = 1,011) (Table 1), the median age was 62 years for both endometrioid adenocarcinoma (EA) and serous cystadenocarcinoma (SC) patients, with the majority aged 51–70. Racial distribution differed significantly between histologies (*p* < 0.01), with SC patients more frequently white and less frequently black compared to EA. Survival outcomes also differed markedly, with a higher proportion of EA patients alive at last follow-up (87%) compared to SC patients (46–51%; *p* < 0.01). SC patients were more likely to have received treatment (55% vs. 37–38% in EA; *p* < 0.01). In sub dataset, treatment type varied by histology: EA patients received both pharmaceutical (52%) and radiation therapy (48%), whereas SC patients received primarily pharmaceutical therapy (100%; *p* < 0.01). Overall, these patterns highlight clinically statistical differences between both morphologies that are preserved in the subset with available gene data.

**Table 1:** Descriptive data of the training dataset and the subset with gene data.

Training dataset (*n* = 2427)	EA		SCa		Dataset with gene data (*n* = 1011)	EA		SC		*p* value
Age(median)	*n* = 796 (62)	%	*n* = 1388 (62)	%	Age(median)	*n* = 369 (62)	%	*n* = 401(62)	%	*p* < 0.05
31–50	**92**	12	262	19	**31–50**	**41**	11	**69**	17	
51–70	512	64	784	56	**51–70**	243	66	228	57	
71–90	192	24	342	25	**71–90**	85	23	104	26	
Race	*n* = 986	%	*n* = 1322	%	Race	*n* = 584	%	*n* = 380	**%**	*p* < 0.01
White	750	76	1128	85	White	444	76	309	81	
American Indian or Alaska native	6	1	8	1	American Indian or Alaska native	2	0	4	1	
Asian	36	4	44	3	Asian	20	3	14	4	
Black or African American	136	14	138	10	Black or African American	67	11	52	14	
Native Hawaiian or other Pacific Islander	16	2	4	0	Native Hawaiian or other Pacific Islander	6	1	1	0	
Other	42	4	0	0	Other	45	8	0	0	
Status	*n* = 1031	%	*n* = 1396	%	Status	*n* = 596	%	*n* = 401	%	*p* < 0.01
Alive	901	87	644	46	Alive	518	87	206	51	
Dead	130	13	752	54	Dead	78	13	195	49	
TTT	*n* = 775	%	*n* = 1342	%	TTT	*n* = 360	%	*n* = 384	%	*p* < 0.01
No	484	62	614	46	No	227	63	172	45	
Yes	291	38	728	55	Yes	133	37	212	55	
TTT type	*n* = 802	%	*n* = 1388	%	TTT type	*n* = 372	%	*n* = 201	%	*p* < 0.01
Pharmaceutical Therapy	401	50	694	50	Pharmaceutical Therapy	193	52	200	100	
Radiation Therapy	401	50	694	50	Radiation Therapy	179	48	1	0	

*EA* Endometriod adenocarcinoma, *SC* Serous Cystadenocarcinoma, *TTT* treatment

### Comparative performance of classification models in predicting outcomes

**Figure 1. ROC curves for the four models applied:**
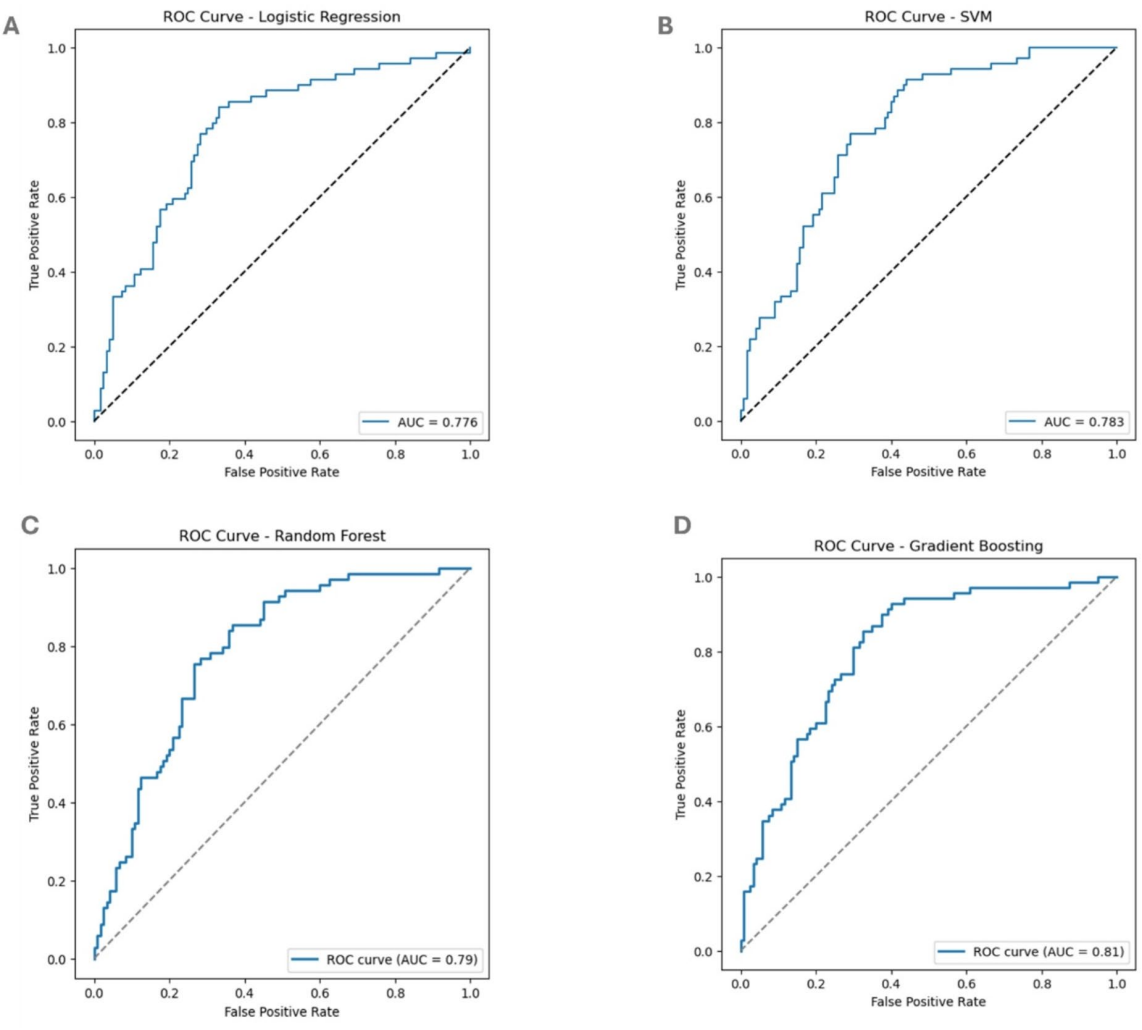
(**A**) Logistic Regression, (**B**) SVM, (**C**) Random Forest and (D) Gradient Boosting. Highest values were observed with GB followed by Random forest, SVM then logistic regression. While Gradient Boosting achieved the highest ROC-AUC (0.81), SVM demonstrated superior recall for mortality cases (0.70 vs. 0.61), which was prioritized given our clinical objective.

**Figure 2. SHAP Analysis Reveals Top Gene Predictors of Mortality Across Machine Learning Models:**
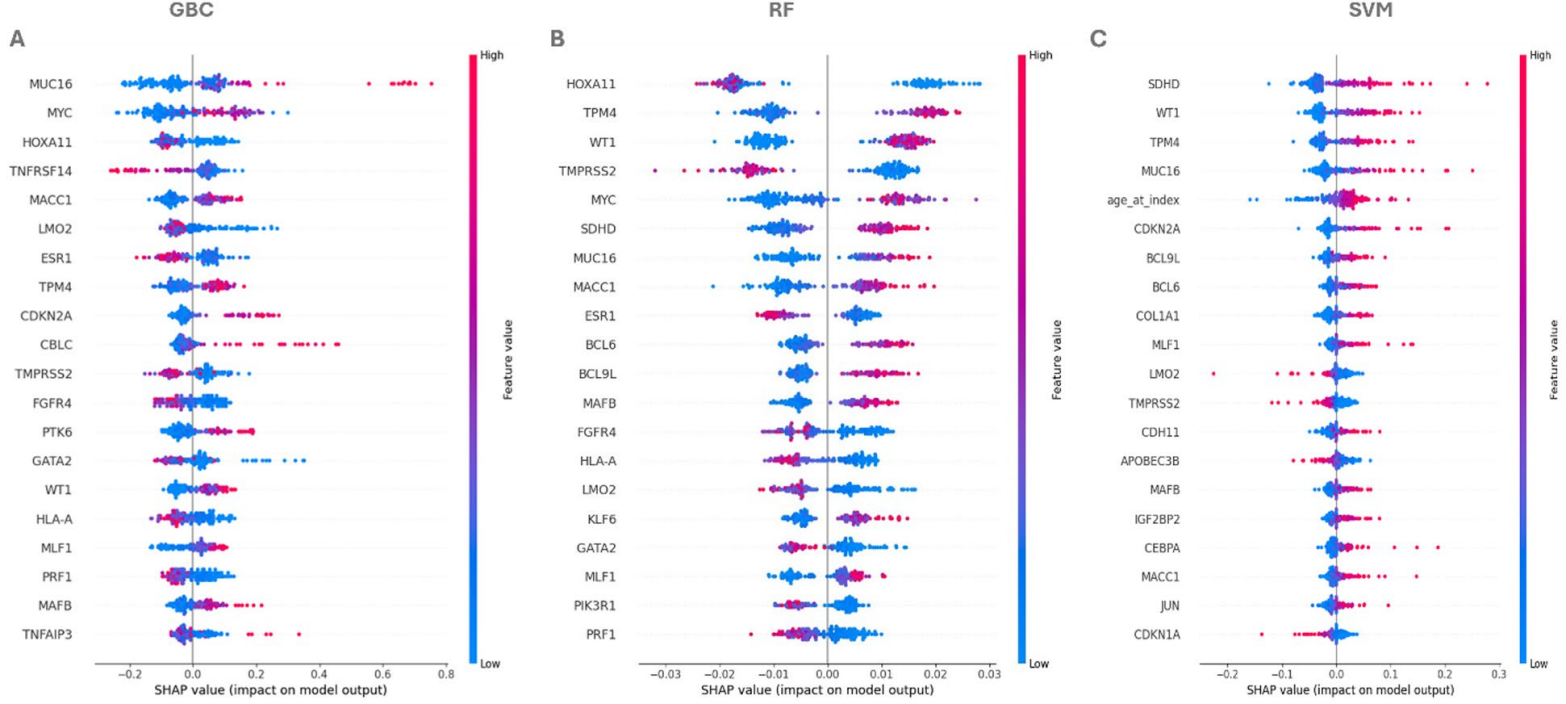
SHAP (Shapley Additive Explanations) analysis of three machine learning models—(**A**) Gradient BoostingClassifier, (**B**) Random Forest, and (**C**) Support Vector Machine—identified key genes predicting two-yearsurvival outcomes in epithelial ovarian cancer. Each panel displays gene features ranked by importance, withblue coloring indicating lower expression values associated with reduced mortality risk and red/pink coloringindicating higher expression values associated with increased mortality risk. Across all three models, WT1,TPM4, HOXA11, TMPRSS2, and MUC16 consistently rank among the top predictors, with SDHD and age atdiagnosis emerging as additional significant contributors in the SVM model. The width and distribution ofSHAP values across each gene row illustrate the consistency and variability of predictive contributions, whilegenes such as MUC16, MACC1, ESR1, BCL6, and LMO2 show moderate but consistent effects with variabledirections. This multi-model consensus highlights robust biomarkers for personalized mortality riskstratification in ovarian cancer.

Our data has shown that SVM was the most effective model using an RBF kernel and logistic regression considering mortality-class recall and F1-score as primary metrics. The distinction between SVM and random forest was minimal, suggesting it might not significantly impact the results. Although GBC demonstrates excellent performance, it does not accurately predict the mortality feature (1) versus survival (0), leading to its exclusion having the lowest F1-score (Table 2; Figure. 1). Given these model performance results, we further investigated the models’ feature importance using SHAP analysis to gain deeper insights into the factors influencing predictions. While SHAP analysis of the linear regression model was inconclusive (data not shown), the other three models consistently identified a shared set of gene expression features as the strongest predictors of survival, with only minor variation in their relative rankings. HOXA11, TPM4, WT1, and TMPRSS2 were highlighted as top drivers in both the Gradient Boosting and Random Forest models (Figure. 2A-B), where higher expression levels were generally associated with increased predicted risk, while lower expression appeared protective. In the SVM model, the most influential features included SDHD, WT1, TPM4, MUC16, and age_at_index, reinforcing the central role of certain genetic markers while also emphasizing the contribution of a clinical feature (patient age) (Figure. 2C). Notably, MUC16, MACC1, ESR1, BCL6, and LMO2 showed moderate but consistent contributions across models, although their effects were sometimes context-dependent, with mixed SHAP patterns suggesting interactions with other features. Taken together, the analyses converge on WT1, TPM4, HOXA11, TMPRSS2, and MUC16 as the most influential gene expression markers across models, while features such as BCL6, LMO2, and MACC1 require additional data and context to clarify their variable effects. These findings underscore that integrating gene expression with clinical data enhances predictive performance and highlights a subset of genes as robust candidates for further investigation.

**Table 2:** Classification matrix for prediction models used for training.

Class	Precision	Recall	F1-score	Algorithm	AUC
0	0.81	0.72	0.77	Logistic regression	0.776
1	0.60	0.71	0.65	Logistic regression	0.776
0	0.81	0.74	0.77	SVM	0.783
1	0.61	0.70	0.65	SVM	0.783
0	0.82	0.73	0.78	Random forest	0.79
1	0.61	0.72	0.66	Random forest	0.79
0	0.78	0.80	0.79	GBC	0.81
1	0.64	0.61	0.62	GBC	0.81

Class 0 = alive at last follow-up; Class 1 = deceased. SVM was selected as the optimal model based on superior recall for mortality class (0.70) and balanced F1-score (0.65), prioritizing accurate identification of high-risk patients. *AUC–ROC* area under the receiver operating characteristic curve.

### Differential gene expression and enrichment analyses

To further explore clinical context and visualize potential subgroup-specific effects, we performed heatmap and volcano plot analyses stratified by clinical variables. Heatmap analysis of the full cohort revealed non-specific patterns in the analyzed subtypes (Figure S2). Based on prior evidence identifying older age and advanced FIGO stage as predictors of poor prognosis, samples were subsequently stratified by age and by FIGO stage and volcano plots differential gene expression among the groups.

**Figure 3. Volcano plot showing the differentially expressed genes in deceased patients with EA:**
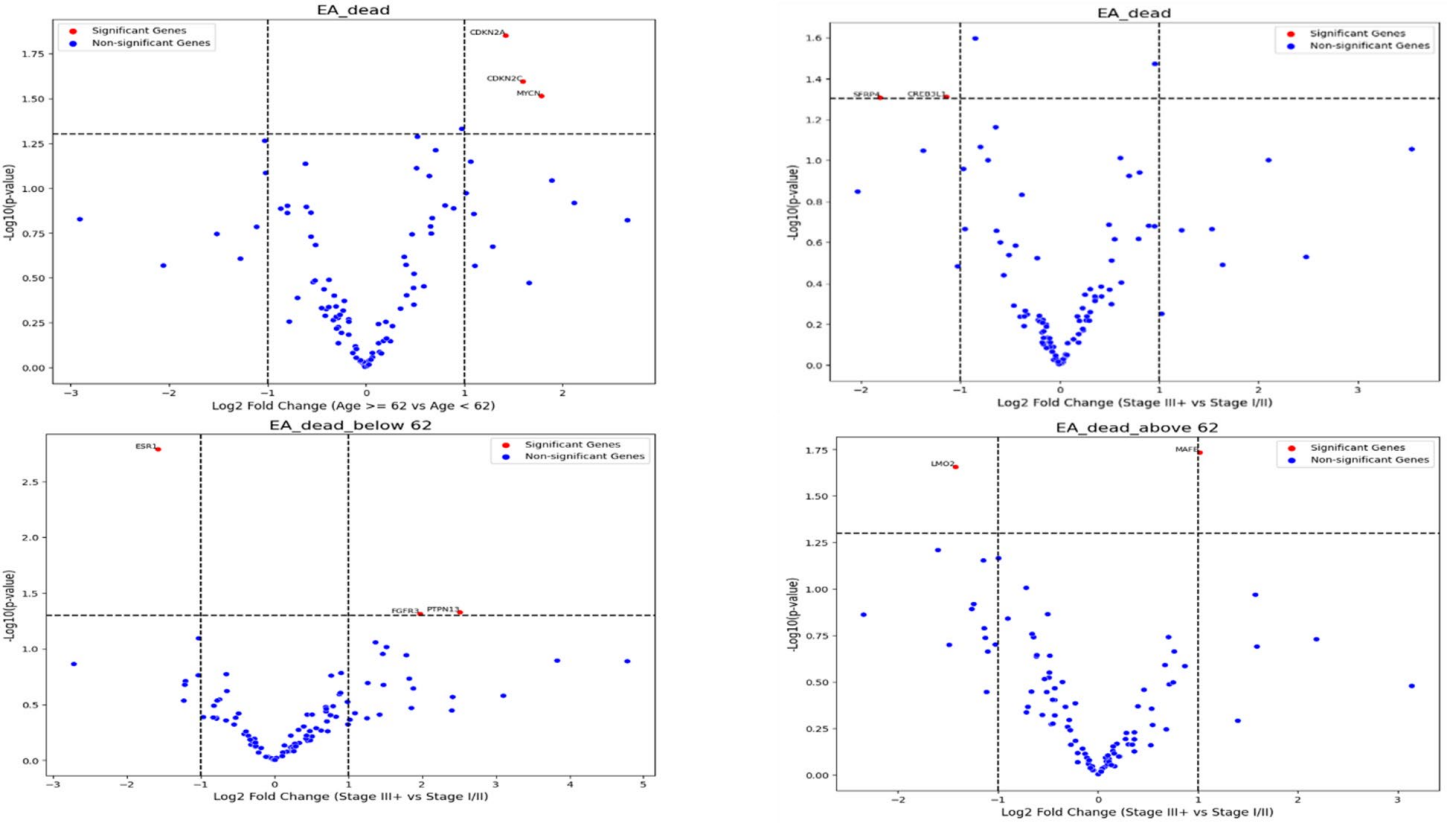
Samples were grouped by age a then by stage (upper panel). Then deceased patients above or below the age of 62 years old were grouped by stages, those with stage III and above versus those below Stage III (lower panel).

**Figure 4. Volcano plot shows the differentially expressed genes in deceased patients with SC:**
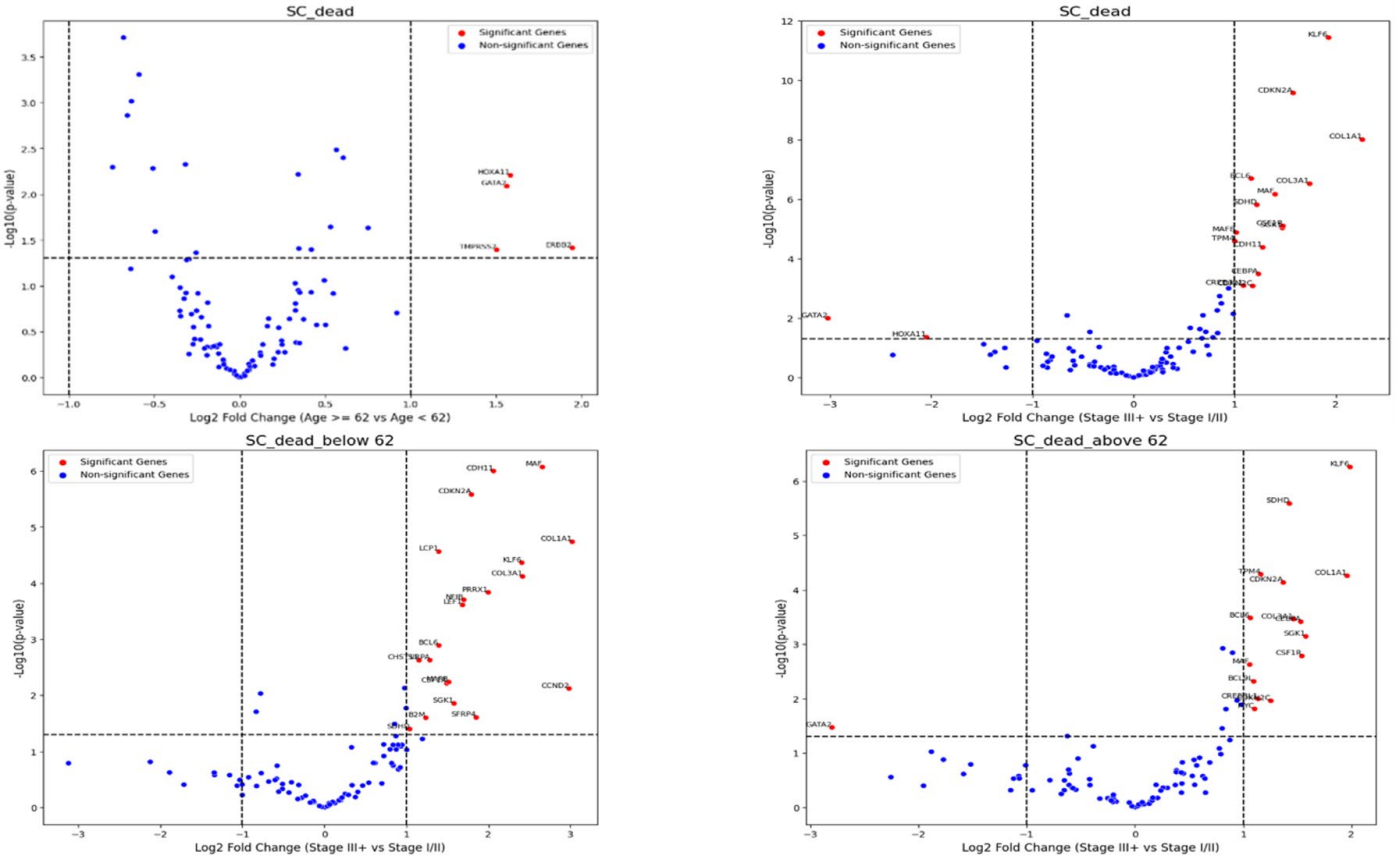
Samples were grouped by age a then by stage (upper panel). Then deceased patients above or below the age of 62 years old were grouped by stages, those with stage III and above versus those below Stage III (lower panel).

Significant differential gene expression patterns, between age groups (< 62 years vs. ≥ 62 years) were observed in deceased patients (*p* < 0.05). The most pronounced upregulations were observed in **PTPN13** (2.51-fold), **CDH11** (2.06-fold), and **CCND2** (2.03-fold), while **GATA2** showed the strongest downregulation (-2.79-fold), followed by **ESR1** (-1.57-fold). Notably, several genes involved in cell cycle regulation (**CCND2**,** CDKN2A/C**), tumor microenvironment (**COL3A1**, **COL1A1**), and growth factor signaling (**FGF18**, **ERBB2**) showed significant upregulation with fold changes ranging from 1.5 to 2.0. The most statistically significant changes were observed in **KLF6** (*p* < 0.001), **SDHD** (*p* < 0.001), and **CDH11** (*p* < 0.001), emphasizing age/grade specific molecular profiles that could influence disease progression and therapeutic responses (Figures. 3 and 4).

Volcano plot analyses revealed distinct gene expression shifts associated with age and FIGO stage in deceased patients. In EA, higher expression of **CDKN2A/C** and **MYCN** was more frequent in older patients (> 62 years), while **FGFR3** and **PTPN3** were enriched in advanced-stage (FIGO III) disease. Conversely, **LMO2** showed lower expression in older and late-stage EA patients. In SC, decreased expression of **GATA2** and **HOXA11** contrasted with higher expression of **KLF6**,** SDHD**,** COL1A1**,** TPM4**,** CDKN2A**,** BCL6**,** MAF**,** MYC**,** CSF1B**,** CREB3L1**,** and CDKN2C** in advanced stage and older patients.

Volcano plots and SHAP analyses identified overlapping but partially distinct gene sets. Genes such as TPM4, SDHD, MYC, CDKN2A/C, and HOXA11 were highlighted by both approaches, whereas several volcano-identified genes (PTPN3, FGFR3, MYCN, LMO2, KLF6, CREB3L1) were not among the top SHAP predictors. Conversely, SHAP ranked WT1, TMPRSS2, MUC16, and MACC1 highly, despite their less pronounced differential expression in volcano analyses. These findings indicate both concordant and divergent molecular signals between univariate and model-based predictive approaches.

**Figure 5. Dot plot showing the enrichment analysis of pathways and gene ontologies:**
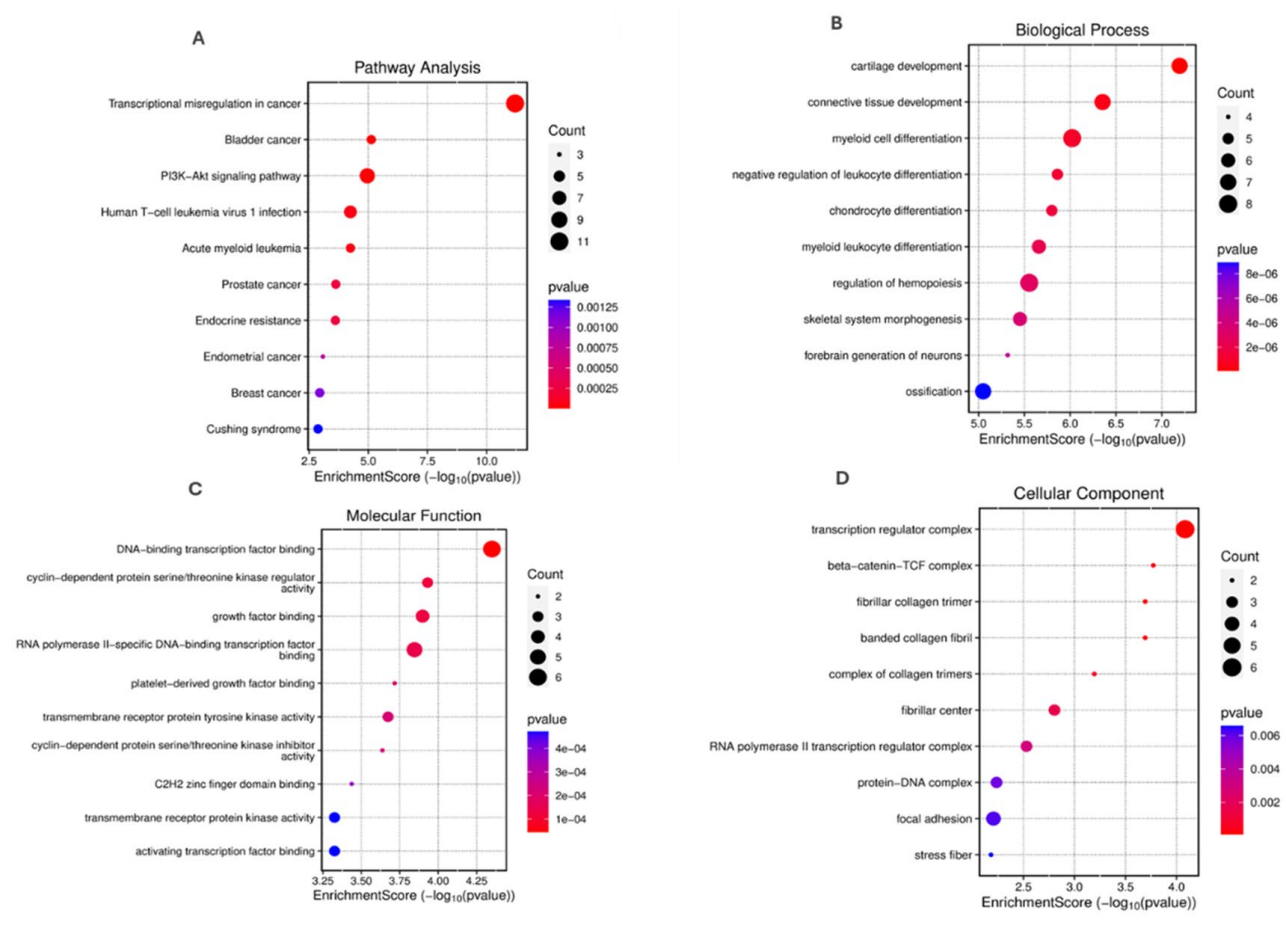
(**A**) Top 10 enriched pathways showing enrichment scores and gene counts. (**B**) Biological process enrichment analysis displaying developmental and cellular differentiation processes. (**C**) Molecular function enrichment analysis highlighting transcription and kinase-related activities. (**D**) Cellular component enrichment analysis showing structural and regulatory complexes. In all panels, dot size represents gene count, color intensity indicates p-value significance, and x-axis shows enrichment score (-log10(p-value)).

To contextualize these gene-level changes within broader biological processes, pathway and gene ontology enrichment analyses were performed. Pathway enrichment analysis revealed significant involvement of transcriptional mis regulation (*p* = 2.33E-12), PI3K/AKT signaling (*p* = 1.11E-05), and other cancer-related pathways, including bladder, breast, gastric, and pancreatic cancer. Additional enrichment was observed in MAPK and mTOR signaling, immune and inflammatory processes, metabolic regulation, and cell adhesion. Gene Ontology (GO) analysis further highlighted enrichment in developmental and differentiation pathways, transcriptional regulation, kinase activity, extracellular matrix organization, and key transcriptional complexes. Full enrichment results are provided in Figure. 5A–D.

### Mutation profiling and survival outcomes in SC and EA

To complement the gene expression analyses and SHAP–volcano comparisons, we next examined mutational profiles and their association with survival outcomes. While the expression-based models were trained on 100 genes (representing all available gene expression measurements on the TCGA for our specific cohort), mutational profiling data were available for a separate panel of 50 genes across 693 patients (Dataset 4). Because many samples had missing mutation calls, these variables were not included in the model training; instead, they were analyzed independently to provide additional context on genomic alterations influencing survival. The mutation frequency analysis reveals significant differences between EA and SC in terms of gene mutations (Table S2).**TP53** mutations are highly prevalent in SC, occurring in 95% of cases compared to only 26% in EA. Other genes, such as **PIK3CA**, show higher mutation rates in SC (77%) compared to EA (59%). Additionally, genes like **ZFHX3**, **FAM135B**, **CSMD3**, and **NF1** demonstrate much higher mutation frequencies in SC, with rates exceeding 70%, whereas their prevalence in EA is notably lower. Conversely, **PTEN** and **ARID1A** are more commonly mutated in EA (91% and 65%, respectively) than in SC (49% for both genes). **PIK3R1** mutations are more frequent in EA (46%) compared to SC (56%). Genes such as **PIK3CA**, **FAT1**, **FAT4**, and **CTNNB1** exhibit relatively similar mutation frequencies across both cancers, though slightly higher in SC for **PIK3CA** and **FAT1/FAT4**. Finally, some genes like **MED12**, **BCORL1**, **ATRX**, **IRS4**, and **FLNA** show low mutation frequencies in SC (3% or lower) but slightly higher rates in EA (up to 19%). These findings highlight the distinct genetic landscape of SC and EA, with certain mutations more strongly associated with one cancer type over the other.

**Figure 6. Mean survival difference between patients with mutated versus non-mutated genes per subtype:**
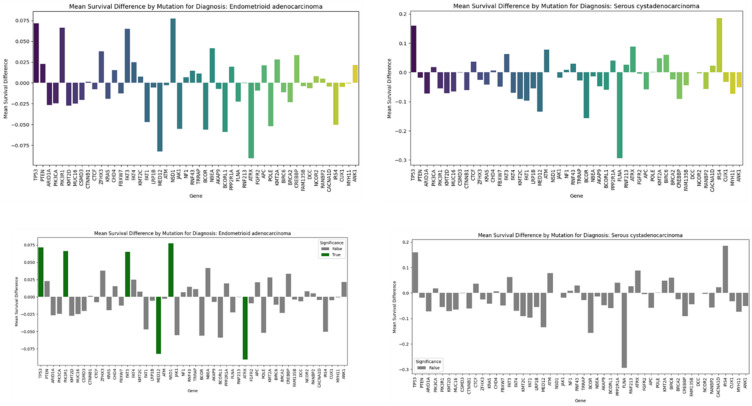
Mean survival difference between patients with mutated versus non-mutated genes per subtype. Positive differences are linked to increased contribution to survival while negative ones are associated with reduced contribution survival.

In EA, patients with mutations in TP53, PIK3R1 and FAT3 NSD1demonstrated a minimal positive significant mean survival difference while minimal negative survival difference was reported with MED12 and ATRX that was statistically significant as well (Figure. 6, left panel). For SC, however, no statistically significant difference was reported between the mutated versus non-mutated (Figure. 6, right panel).

**Figure. 7:**
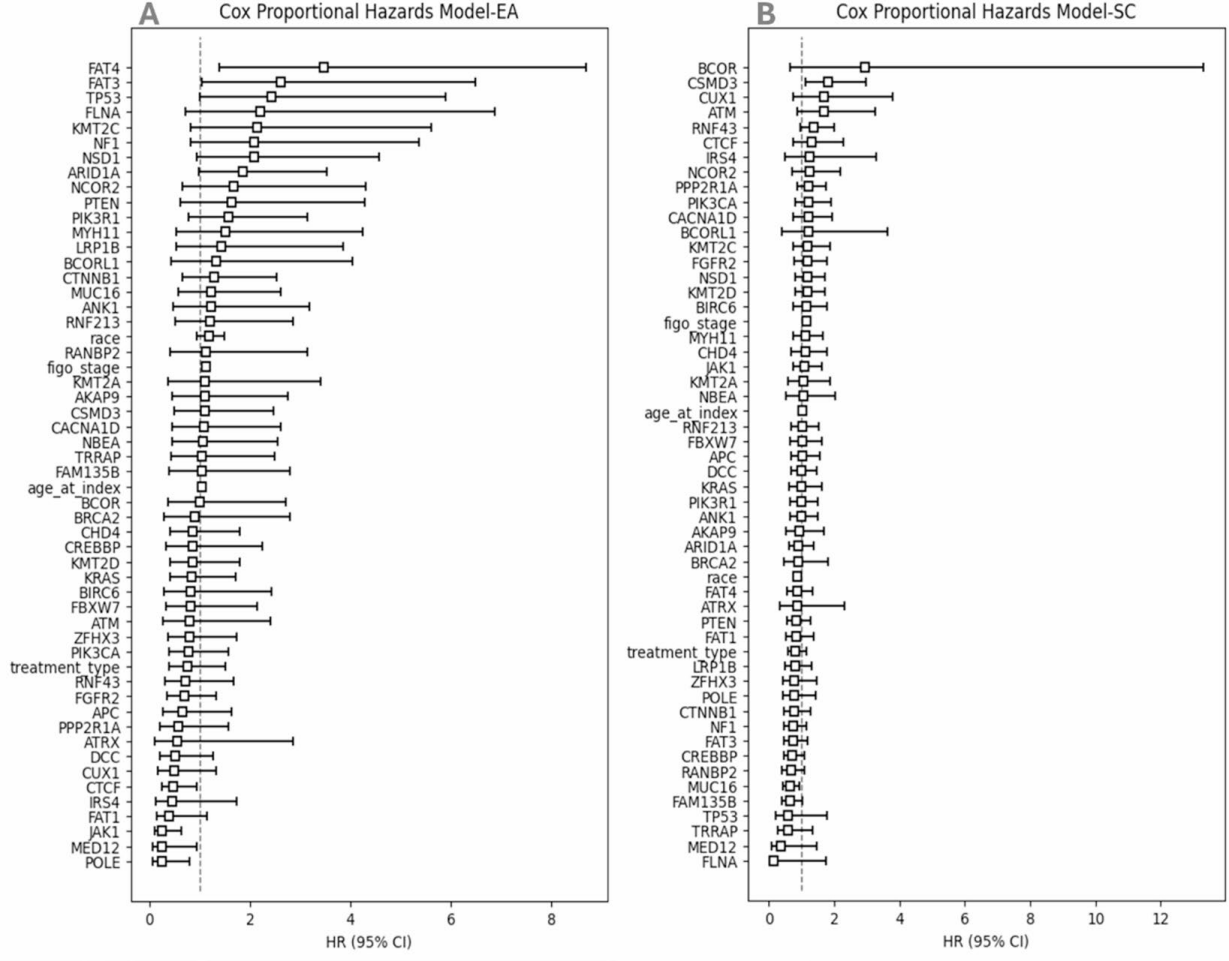
Cox Proportional Hazards Analysis of Clinical and Genetic Prognostic Factors in EA and SC. Similar genes had different HR in each group.

Cox proportional hazards analysis was conducted on both groups; patients with EA, and SC (Figure. 7). In EA, a higher FIGO stage was significantly associated with an increased hazard of death (HR = 1.12, 95% CI: 1.06–1.18, *p* < 0.0001), indicating that advancing disease stage worsens prognosis. Similarly, older age at diagnosis was linked to a slightly increased risk of death (HR = 1.04, 95% CI: 1.02–1.05, *p* < 0.05). Among genetic factors, the presence of **TP53** mutation was associated with a higher hazard of death (HR = 2.43, 95% CI: 1.00–5.89, *p* < 0.05), suggesting its role as a poor prognostic indicator. Conversely, **CTCF** mutation demonstrated a protective effect, significantly reducing the risk of death (HR = 0.48, 95% CI: 0.24–0.97, *p* < 0.05) (Figure. 7, upper panel).

Additionally, **FAT3** (HR = 2.60, 95% CI: 1.05–6.48, *p* < 0.05) and **FAT4** (HR = 3.46, 95% CI: 1.38–8.72, *p* < 0.05) mutations were both associated with significantly increased hazards of death, highlighting their potential contribution to poor outcomes. In contrast, mutations in **MED12** (HR = 0.24, 95% CI: 0.06–0.94, *p* < 0.05), **JAK1** (HR = 0.25, 95% CI: 0.10–0.64, *p* < 0.05), and **POLE** (HR = 0.24, 95% CI: 0.07–0.72, *p* < 0.05) were associated with significantly decreased risks of death, indicating their potential protective roles (Figure. 8, middle panel)(Table S3). These results underscore the prognostic significance of both clinical and genetic factors in influencing survival outcomes.

**Figure 8. Kaplan Meier curves showing the survival pattern for the patients with affected (orange lines) versus non-affected genes (blue lines):**
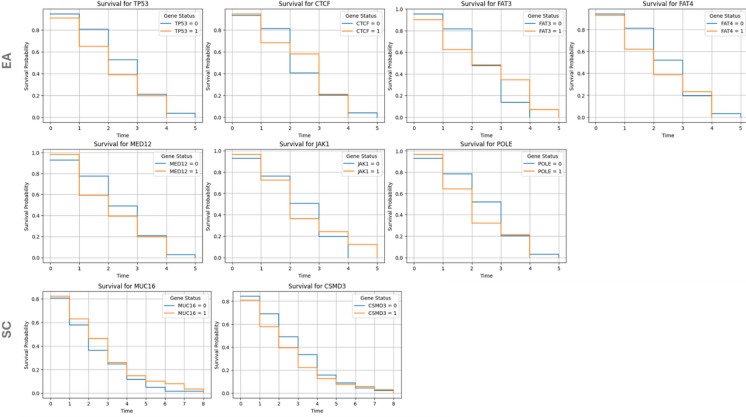
The number of patients per gene is included in Table S4.

For SC, a higher FIGO stage was significantly associated with an increased risk of death (HR = 1.14, 95% CI: 1.07–1.21, *p* < 0.0001), reinforcing its role as a critical prognostic factor. Race showed a protective effect, with a reduced hazard of death (HR = 0.87, 95% CI: 0.78–0.96, *p* < 0.05), indicating potential disparities in survival outcomes that warrant further investigation. Age at diagnosis was also significantly associated with an increased hazard of death (HR = 1.04, 95% CI: 1.02–1.05, *p* < 0.0001), consistent with findings from prior analyses. Regarding genetic factors, the presence of **MUC16** mutation was associated with a decreased hazard of death (HR = 0.64, 95% CI: 0.43–0.93, *p* < 0.05), suggesting its potential protective role. In contrast, **CSMD3** mutation was significantly associated with an increased hazard of death (HR = 1.81, 95% CI: 1.10–2.98, *p* < 0.05), indicating its potential as a marker of poor prognosis (Figure. 8, lower panel). For all the cohort (*n* = 997), median survival years were detected to **be 2 years** for most of the genes with statistically significant values reported with FBXW7, FAT4 and FAT1and 3 years with ATRX mutations (*p* < 0.05) (Table S5) with only 12 samples showing more than 5-year survival (data not shown). These findings further underscore the importance of integrating clinical and genetic factors in assessing survival outcomes.

## Discussion

Integrating transcriptomic and genomic data with clinical features through machine learning offers a powerful approach to predict survival and uncover prognostic biomarkers. Epithelial ovarian cancer (EOC) exhibits marked heterogeneity in clinical outcomes, influenced by histology, age, stage, and molecular characteristics^17^. Understanding how gene expression and mutational profiles interact with clinical factors is critical for improving prognostic prediction and guiding personalized therapies. Such integrative models can capture complex, context-dependent interactions that traditional analyses may overlook. In our study, we aimed at integrating multiomic parameters with clinical and demographics into outcome prediction machine learning models, then complemented these analyses with biological interpretation for enhanced personalized risk stratification that guides treatment decision-making in EOC (Figure S3). Our cohort included 2,427 patients with EOC subtypes; Endometrioid Adenocarcinoma (EA) and Serous cystadenocarcinoma (SC), of whom 1,011 had gene expression data. The cohort EA and SC subtypes, with a median age of 62 years. Significant differences were observed between histologies: EA patients exhibited higher survival rates and more balanced treatment modalities, whereas SC patients had lower survival and predominantly received pharmaceutical therapy. These findings are consistent with prior studies showing histology-specific survival outcomes and treatment responses^18,19^.

Age and FIGO stage were strong clinical predictors of survival, reinforcing the importance of integrating patient demographics and tumor characteristics with molecular data for robust prognostic modeling^20,21^.

Among the tested models, Gradient Boosting Classifier (GBC), Support Vector Machines (SVM) and Random Forest outperformed logistic regression, with SVM showing slightly superior predictive accuracy. While GBC achieved the highest AUC (0.81), it was deprioritized because it did not align as closely with the study’s focus on interpretability and mortality prediction. Scaling using robust scalers effectively handled non-normal distributions and outliers in gene expression data.

SHAP analyses revealed that WT1, HOXA11, TPM4, TMPRSS2, MUC16, SDHD, and MYC were the most influential predictors of mortality. Age at diagnosis was also a strong contributor. Higher values of these features generally increased predicted risk, while lower values favored survival. Genes lower in the ranking, such as JUN, MACC1, and CDKN1A, contributed modestly, indicating a hierarchical structure of gene-level influence on survival. Comparison of SHAP rankings with volcano plot-derived differential expression highlighted both overlap and divergence. Genes such as TPM4, SDHD, MYC, CDKN2A/C, and HOXA11 were consistently identified by both approaches, reflecting their dual role as stage- and age-associated markers and strong survival predictors. In contrast, genes prominent in volcano plots; PTPN3, FGFR3, MYCN, LMO2, KLF6, CREB3L1, were less predictive at the patient level, suggesting context-specific effects that may not generalize across patients. Conversely, SHAP identified WT1, TMPRSS2, MUC16, and MACC1 as major survival drivers, despite minimal differential expression. This underscores that integrating univariate expression analyses with multivariate predictive modeling captures both patient-level and group-level prognostic signals. Several top SHAP-identified genes have well-characterized mechanistic roles in ovarian cancer. WT1 promotes epithelial-mesenchymal transition (EMT) via E-cadherin modulation and ERK1/2 signaling^22^, in addition to HOXA11 acting as a tumor promoter in EOC^23^ while TPM4 drives EMT, metastatic dissemination, and chemotherapy resistance^24^. Dysregulation of SDHD sensitizes cyclin E-driven ovarian cancers to CDK inhibition^25^. MUC16 contributes to tumor proliferation, migration, and immune evasion, representing a potential immunotherapy target^26^;^27^. Similarly, BCL6^28^and BCL9^29^ facilitate tumor progression and are potential therapeutic targets, and CDKN2A promoter methylation correlates with poorer progression-free survival, supporting its prognostic significance^30^. Other genes, including LMO2^31^, COL1A1^32^, MACC1^33^, IGF2BP1^34^, TMPRSS2^35^CEBPA^36^, JUN^37^and MAFB^38^, are involved in invasiveness, EMT, stemness, and immune microenvironment remodeling, illustrating the complex networks underlying ovarian cancer progression.Mutational analyses revealed distinct histology-specific patterns: SC showed high mutation rates in TP53^39^, PIK3CA^40^, and ZFHX3^41^, whereas EA had higher prevalence of PTEN and ARID1A mutations^42,43^. Several mutations were associated with survival: TP53^17,44^, FAT3^45^, and FAT4^46^ increased hazard in EA, while CSMD3 increased hazard in SC^47^. Conversely, MUC16 appeared protective. Median survival for significantly mutated genes ranged from 2 to 3 years, emphasizing their clinical relevance.

Integrating mutation profiles with gene expression and clinical data enhances prognostic modeling, demonstrating that survival outcomes in EOC arise from a multifactorial interplay of patient, transcriptomic, and genomic features. Our findings align with recent machine learning–based ovarian cancer studies. Gradient boosting models have consistently outperformed conventional Cox regression in survival prediction^18^;^19^, while multi-omics deep learning frameworks, improved risk stratification and therapy guidance^48^. Image-based models also effectively predicted prognosis and therapy response^49^. Our study complements these works by integrating clinical, genomic, and transcriptomic data, providing patient-level predictive insights alongside understanding of key genes. Limitations include incomplete gene expression data (*n* = 1,011) and missing mutational information for some patients, potentially affecting generalizability. Future studies should expand multi-omics integration, incorporate spatial transcriptomics or proteomics, and validate findings prospectively. Functional validation of candidate biomarkers, such a TPM4, SDHD, MUC16, and BCL6 could translate predictive modeling into actionable therapeutic strategies.

## Conclusion

Our study demonstrates that combining clinical, genomic, and transcriptomic data with machine learning improves survival prediction in EOC. SHAP analyses provide patient-level risk insights that complement differential expression and mutation profiling. A group of genes has been identified e as potential prognostic drivers, highlighting avenues for future studies and targeted therapies. Integrating multivariate predictive modeling with biological interpretation enables a comprehensive framework for personalized risk stratification and treatment decision-making in ovarian cancer.

## Supplementary Information

Below is the link to the electronic supplementary material.

Supplementary Material 1

Supplementary Material 2

Supplementary Material 3

Supplementary Material 4

Supplementary Material 5

## Data Availability

The raw data used in this study is publicly available through the TCGA data portal (https://portal.gdc.cancer.gov/analysis_page?app=CohortBuilder&tab=general). Datasets are included. All scripts and codes used to develop the machine learning models are available upon request from the authors.
