# Supplementary material for: Integrating multi-omics and clinical features to model survival in epithelial ovarian cancer subtypes
Source: Sci Rep. 2025 Dec 19;15:44221. doi: 10.1038/s41598-025-29403-6 (PMC12722313; doi:10.1038/s41598-025-29403-6)
Supplement: Supplementary file 5 — Supplementary Material 5 [file 41598_2025_29403_MOESM5_ESM.pdf]

Table S1. Genes used in study analyses

Analysis Category	Gene
**Cox**	AKAP9, ANK1, APC, ARID1A, ATM, ATRX, BCOR, BCORL1, BIRC6, BRCA2, CACNA1D, CHD4, CREBBP, CSMD3, CTCF, CTNNB1, CUX1, DCC, FAM135B, FAT3, FAT4, FBXW7, FLNA, IRS4, JAK1, KMT2A, KMT2C, KMT2D, KRAS, LRP1B, MED12, MYH11, NBEA, NCOR2, NF1, NSD1, PIK3CA, POLE, PPP2R1A, PTEN, RANBP2, RNF213, RNF43, TRRAP, and ZFHX3
**Differential**	JUN, ACKR3, ALDH2, APOBEC3B, ATP1A1, AXIN2, B2M, BCL3, BCL6, BCL9L, BIRC3, BTG2, CARD11, CBLC, CCND1, CCND2, CCNE1, CD74, CD79A, CDH1, CDH11, CDKN1A, CDKN1B, CDKN2A, CDKN2C, CEBPA, CHST11, COL1A1, COL3A1, CREB3L1, CSF1R, CXCR4, ELF3, ELN, ERBB2, ERBB3, ERBB4, ESR1, ETV4, ETV5, FBLN2, FGFR1, FGFR3, FGFR4, FSTL3, GATA2, HLA-A, HMGA1, HOXA11, ID3, IGF2BP2, KLF6, LCP1, LEF1, LMO2, MACC1, MAF, MAFB, MECOM, MET, MLF1, MLLT11, MUC1, MYC, MYCL, MYCN, NDRG1, NFIB, PAX8, PCBP1, PDGFRA, PDGFRB, POU5F1, PREX2, PRF1, PRRX1, PTK6, PTPN13, SDHD, SFRP4, SGK1, SIRPA, SIX1, SLC34A2, SOCS1, TBX3, TENT5C, TFRC, TMPRSS2, TNC, TNFAIP3, TNFRSF14, TPM4, WNK2, and WT1.
**Differential & Cox**	FAT1, FGFR2, MUC16, PIK3R1, and TP53.

Table S2. Mutation frequency in EA versus SC.

Gene	SC (n=401)	% of SC cases	EA(n=596)	% of EA cases	*p*_value	Total Samples with reported information on the gene
TP53	383	95	146	26	6.0148E-107	529
PIK3CA	310	77	333	59	6.53476E-12	643
ZFHX3	288	72	234	41	1.14218E-23	522
FAM135B	287	71	195	34	4.92324E-33	482
CSMD3	286	71	244	43	7.81803E-21	530
NF1	276	69	147	26	3.87858E-43	423
CTCF	271	67	253	44	1.0961E-14	524
MUC16	255	63	201	35	3.05022E-20	456
FAT1	253	63	156	27	6.97025E-31	409
RNF213	250	62	153	27	1.25263E-30	403
KMT2C	246	61	191	34	1.11062E-19	437
FBXW7	236	59	132	23	1.13655E-31	368
NBEA	231	57	148	26	2.84683E-25	379
CHD4	227	56	162	28	1.78936E-20	389
BRCA2	227	56	133	23	4.41447E-28	360
FAT4	226	56	154	27	4.32494E-22	380
DCC	226	56	135	24	3.758E-27	361
PIK3R1	225	56	262	46	0.000216707	487
CREBBP	222	55	134	24	4.72131E-26	356
PPP2R1A	220	55	102	18	1.80398E-35	322
RNF43	218	54	170	30	3.967E-16	388
ANK1	210	52	178	31	1.44371E-12	388
KRAS	208	52	195	34	2.27728E-09	403
ATM	208	52	155	27	1.51892E-16	363
KMT2A	208	52	131	23	2.98234E-22	339
FAT3	205	51	169	30	5.44262E-13	374
CUX1	202	50	157	28	1.54237E-14	359
AKAP9	201	50	167	29	2.13779E-12	368
ARID1A	198	49	372	65	5.95265E-05	570
PTEN	197	49	515	91	5.60753E-37	712
TRRAP	197	49	173	30	1.82942E-10	370
NCOR2	196	49	146	26	3.17729E-15	342
POLE	195	49	152	27	9.43976E-14	347
APC	193	48	129	23	3.30931E-18	322
MYH11	192	48	115	20	1.78001E-21	307
FGFR2	190	47	224	39	0.002587622	414
NSD1	189	47	160	28	7.16246E-11	349
BIRC6	185	46	155	27	7.73144E-11	340
KMT2D	181	45	212	37	0.003027405	393
LRP1B	163	41	150	26	3.48854E-07	313

CACNA1D	156	39	141	25	3.57113E-07	297
RANBP2	153	38	141	25	1.22555E-06	294
CTNNB1	141	35	243	43	0.085717962	384
JAK1	137	34	146	26	0.001160774	283
MED12	14	3	110	19	4.4145E-12	124
BCORL1	14	3	98	17	4.15882E-10	112
ATRX	14	3	92	16	3.74626E-09	106
IRS4	12	3	80	14	4.54492E-08	92
FLNA	10	2	98	17	7.63699E-12	108
BCOR	9	2	106	19	1.07519E-13	115

Table S3. Cox results for Studied genes

Sub-type	Gene	coef	exp(coef)	se(coef)	coef lower 95%	coef upper 95%	exp(coef) lower 95%	exp(coef) upper 95%	cmp to	z	p	-log2(p)
EA	**TP53**	0.888181	2.430703	0.451521	0.003215	1.773146	1.00322	5.889352	0	1.967085	0.05	4.345977
	**CTCF**	-0.73728	0.478413	0.347927	-1.41921	-0.05536	0.241906	0.946148	0	-2.11907	0.03	4.874729
	**FAT3**	0.956936	2.603707	0.465318	0.044929	1.868943	1.045954	6.481443	0	2.05652	0.04	4.653541
	**FAT4**	1.24233	3.463674	0.468584	0.323923	2.160737	1.382541	8.677529	0	2.651245	0.01	6.962259
	**MED12**	-1.40893	0.244404	0.691602	-2.76445	-0.05342	0.063011	0.947983	0	-2.0372	0.04	4.586241
	**JAK1**	-1.38328	0.250754	0.476714	-2.31762	-0.44894	0.098507	0.638304	0	-2.90171	0	8.073832
	**POLE**	-1.42827	0.239724	0.610643	-2.6251	-0.23143	0.072432	0.7934	0	-2.33895	0.02	5.692432
SC	**MUC16**	-0.4539	0.635145	0.196738	-0.8395	-0.0683	0.431926	0.933978	0	-2.30714	0.02	5.57024
	**CSMD3**	0.594798	1.812665	0.25331	0.09832	1.091276	1.103316	2.978072	0	2.348106	0.02	5.727826

Table S4. Sample Distribution for Kaplan–Meier Analysis.

EA	Years of Survival						
**Tp53**	**0**	**1**	**2**	**3**	**4**	**5**	**Total**
Un affected	10	4	8	9	5	1	37
Affected	4	4	4	3	3		18
**Total**	**14**	**8**	**12**	**12**	**8**	**1**	**55**
**CTCF**	**0**	**1**	**2**	**3**	**4**	**5**	**Total**
Un affected	9	3	10	5	4	1	32
Affected	5	5	2	7	4		23
**Total**	**14**	**8**	**12**	**12**	**8**	**1**	**55**
**FAT3**	**0**	**1**	**2**	**3**	**4**	**5**	**Total**
Un affected	9	4	10	10	4		37
Affected	5	4	2	2	4	1	18
**Total**	**14**	**8**	**12**	**12**	**8**	**1**	**55**
**FAT4**	**0**	**1**	**2**	**3**	**4**	**5**	**Total**
Un affected	9	4	9	10	5	1	38
Affected	5	4	3	2	3		17
**Total**	**14**	**8**	**12**	**12**	**8**	**1**	**55**
**MED12**	**0**	**1**	**2**	**3**	**4**	**5**	**Total**
Un affected	13	6	11	11	7	1	49
Affected	1	2	1	1	1		6
**Total**	**14**	**8**	**12**	**12**	**8**	**1**	**55**
**JAK1**	**0**	**1**	**2**	**3**	**4**	**5**	**Total**
Un affected	12	6	9	11	7		45
Affected	2	2	3	1	1	1	10
**Total**	**14**	**8**	**12**	**12**	**8**	**1**	**55**
**POLE**	**0**	**1**	**2**	**3**	**4**	**5**	**Total**
Un affected	13	5	9	11	6	1	45
Affected	1	3	3	1	2		10
**Total**	**14**	**8**	**12**	**12**	**8**	**1**	**55**

SC	Years of Survival									
**MUC16**	**0**	**1**	**2**	**3**	**4**	**5**	**6**	**7**	**8**	**Total**
Un affected	6	14	13	7	8	4	2		1	55
Affected	23	17	15	18	10	4	2	4	3	96
**Total**	**29**	**31**	**28**	**25**	**18**	**8**	**4**	**4**	**4**	**151**
**CSMD3**	**0**	**1**	**2**	**3**	**4**	**5**	**6**	**7**	**8**	**Total**
Un affected	7	7	9	7	8	3	2	1	1	45
Affected	22	24	19	18	10	5	2	3	3	106
**Total**	**29**	**31**	**28**	**25**	**18**	**8**	**4**	**4**	**4**	**151**

Table S5. Median Survival by Gene Mutation

Gene	Group	Median Survival	Log-Rank p-value	Gene	Group	Median Survival	Log-Rank p-value
TP53	Non-Affected	2	0.868298	FAT4	Non-Affected	2	0.01827
TP53	Affected	2	0.868298	FAT4	Affected	2	0.01827
PTEN	Non-Affected	2	0.788585	KMT2C	Non-Affected	2	0.128042
PTEN	Affected	2	0.788585	KMT2C	Affected	2	0.128042
ARID1A	Non-Affected	2	0.754949	FAT1	Non-Affected	2	0.044229
ARID1A	Affected	2	0.754949	FAT1	Affected	2	0.044229
PIK3CA	Non-Affected	2	0.611688	LRP1B	Non-Affected	2	0.362128
PIK3CA	Affected	2	0.611688	LRP1B	Affected	2	0.362128
PIK3R1	Non-Affected	2	0.738014	MED12	Non-Affected	2	0.121225
PIK3R1	Affected	2	0.738014	MED12	Affected	3	0.121225
KMT2D	Non-Affected	2	0.139832	ATM	Non-Affected	2	0.685269
KMT2D	Affected	2	0.139832	ATM	Affected	2	0.685269
MUC16	Non-Affected	2	0.686797	NSD1	Non-Affected	2	0.572251
MUC16	Affected	2	0.686797	NSD1	Affected	2	0.572251
CSMD3	Non-Affected	2	0.335069	JAK1	Non-Affected	2	0.974008
CSMD3	Affected	2	0.335069	JAK1	Affected	2	0.974008
CTNNB1	Non-Affected	2	0.20288	NF1	Non-Affected	2	0.123142
CTNNB1	Affected	2	0.20288	NF1	Affected	2	0.123142
CTCF	Non-Affected	2	0.252395	RNF43	Non-Affected	2	0.834452
CTCF	Affected	2	0.252395	RNF43	Affected	2	0.834452
ZFHX3	Non-Affected	2	0.805083	TRRAP	Non-Affected	2	0.290399
ZFHX3	Affected	2	0.805083	TRRAP	Affected	2	0.290399
KRAS	Non-Affected	2	0.694183	BCOR	Non-Affected	2	0.819872
KRAS	Affected	2	0.694183	BCOR	Affected	1	0.819872
CHD4	Non-Affected	2	0.908547	NBEA	Non-Affected	2	0.420323
CHD4	Affected	2	0.908547	NBEA	Affected	2	0.420323
FBXW7	Non-Affected	2	0.044557	AKAP9	Non-Affected	2	0.61164
FBXW7	Affected	2	0.044557	AKAP9	Affected	2	0.61164
FAT3	Non-Affected	2	0.76169	BCORL1	Non-Affected	2	0.899067
FAT3	Affected	2	0.76169	BCORL1	Affected	2	0.899067

FAT4	Non-Affected	2	0.01827	PPP2R1A	Non-Affected	2	0.640155
FAT4	Affected	2	0.01827	PPP2R1A	Affected	2	0.640155
KMT2C	Non-Affected	2	0.128042	FLNA	Non-Affected	2	0.213622
KMT2C	Affected	2	0.128042	FLNA	Affected	3	0.213622
FAT1	Non-Affected	2	0.044229	FLNA	Non-Affected	2	0.213622
FAT1	Affected	2	0.044229	FLNA	Affected	3	0.213622
LRP1B	Non-Affected	2	0.362128	RNF213	Non-Affected	2	0.525327
LRP1B	Affected	2	0.362128	RNF213	Affected	2	0.525327
MED12	Non-Affected	2	0.121225	ATRX	Non-Affected	2	0.033041
MED12	Affected	3	0.121225	ATRX	Affected	3	0.033041
ATM	Non-Affected	2	0.685269	FGFR2	Non-Affected	2	0.059272
ATM	Affected	2	0.685269	FGFR2	Affected	2	0.059272
NSD1	Non-Affected	2	0.572251	APC	Non-Affected	2	0.230844
NSD1	Affected	2	0.572251	APC	Affected	2	0.230844
JAK1	Non-Affected	2	0.974008	POLE	Non-Affected	2	0.871613
JAK1	Affected	2	0.974008	POLE	Affected	2	0.871613
NF1	Non-Affected	2	0.123142	KMT2A	Non-Affected	2	0.598338
NF1	Affected	2	0.123142	KMT2A	Affected	2	0.598338
RNF43	Non-Affected	2	0.834452	BIRC6	Non-Affected	2	0.498272
RNF43	Affected	2	0.834452	BIRC6	Affected	2	0.498272
TRRAP	Non-Affected	2	0.290399	BRCA2	Non-Affected	2	0.154535
TRRAP	Affected	2	0.290399	BRCA2	Affected	2	0.154535
BCOR	Non-Affected	2	0.819872	CREBBP	Non-Affected	2	0.057801
BCOR	Affected	1	0.819872	CREBBP	Affected	2	0.057801
NBEA	Non-Affected	2	0.420323	FAM135B	Non-Affected	2	0.82467
NBEA	Affected	2	0.420323	FAM135B	Affected	2	0.82467
AKAP9	Non-Affected	2	0.61164	DCC	Non-Affected	2	0.128523
AKAP9	Affected	2	0.61164	DCC	Affected	2	0.128523
BCORL1	Non-Affected	2	0.899067	NCOR2	Non-Affected	2	0.67973
BCORL1	Affected	2	0.899067	NCOR2	Affected	2	0.67973
PPP2R1A	Non-Affected	2	0.640155	RANBP2	Non-Affected	2	0.797646
PPP2R1A	Affected	2	0.640155	RANBP2	Affected	2	0.797646
CUX1	Non-Affected	2	0.464045	CACNA1D	Non-Affected	2	0.326898

CUX1	Affected	2	0.464045	CACNA1D	Affected	2	0.326898
MYH11	Non-Affected	2	0.387602	IRS4	Non-Affected	2	0.558513
MYH11	Affected	2	0.387602	IRS4	Affected	3	0.558513
ANK1	Non-Affected	2	0.354841				
ANK1	Affected	2	0.354841				

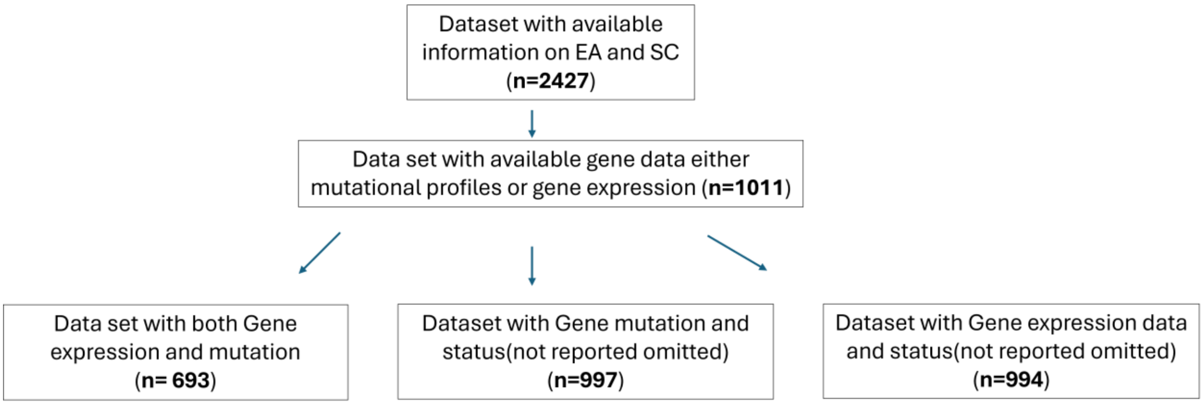

Figure S1. Figure S1. Datasets and sampling.

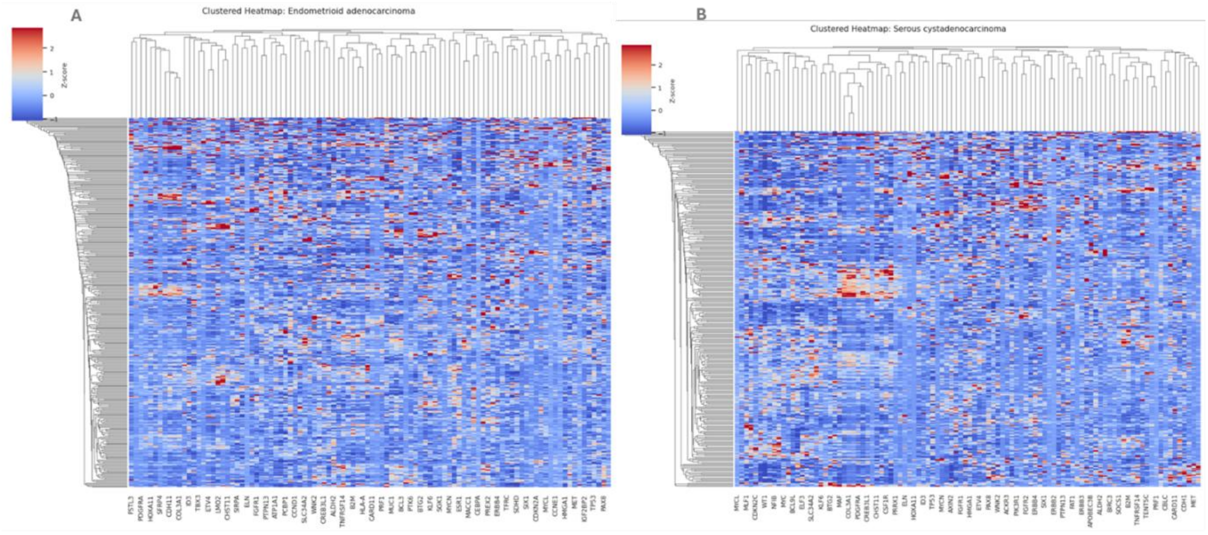

Figure S2. Heat map analysis for the differentially expressed genes in EA versus SC. Non-Specific patterns are observed.

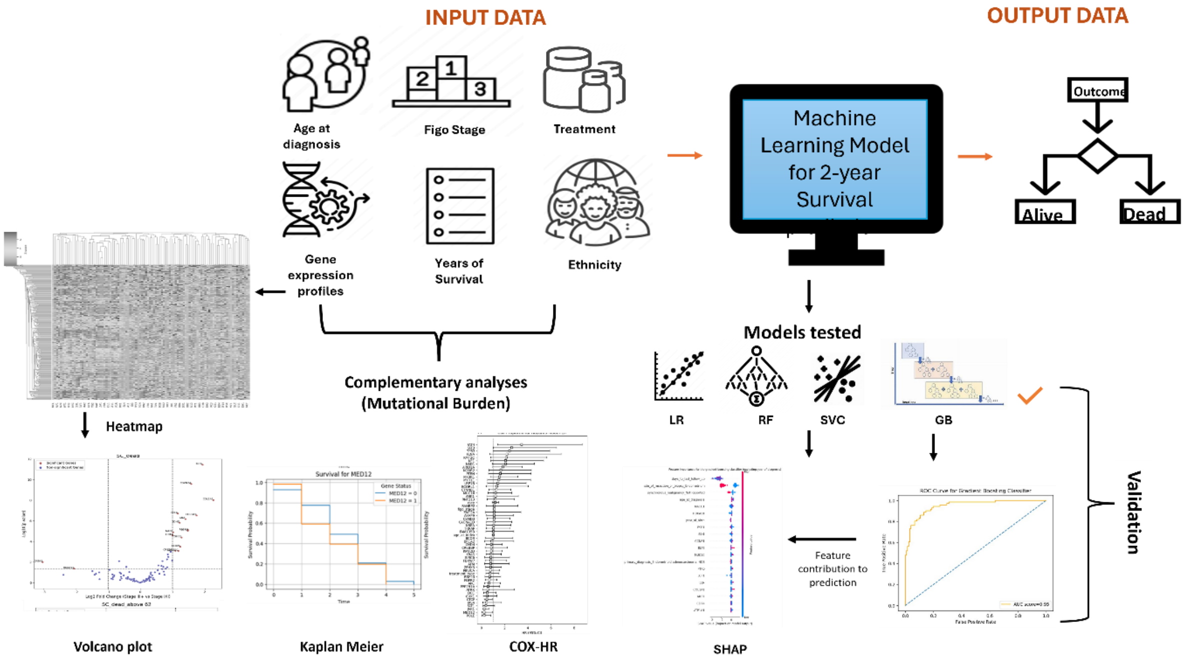

Figure S3. Methodology Summary
